# Supplementary material for: Enhancing surface activity and durability in triple conducting electrode for protonic ceramic electrochemical cells
Source: Nat Commun. 2025 May 4;16:4146. doi: 10.1038/s41467-025-59477-9 (PMC12049472; doi:10.1038/s41467-025-59477-9)
Supplement: Supplementary file 1 — Supplementary Information [file 41467_2025_59477_MOESM1_ESM.pdf]

Supplementary Information

Enhancing Surface Activity and Durability in Triple Conducting Electrode for Protonic Ceramic Electrochemical Cells

Shuanglin Zheng[1], Wei Wu[2], Yuchen Zhang[2], Zeyu Zhao[2], Chuancheng Duan[3], Saroj Karki[1], Hanping Ding[1,*]

[1] School of Aerospace and Mechanical Engineering, University of Oklahoma, Norman, OK 73019 USA.

[2] Energy and Environment Science & Technology, Idaho National Laboratory, Idaho Falls, ID 83415 USA.

[3] Department of Chemical Engineering, University of Utah, Salt Lake City, UT 84112 USA

Correspondence authors' email: hding@ou.edu

This PDF file includes:

Equation S1
Figs. S1 to S11
Tables S1 to S19

Equation. S1.

Equation. S1**.** The relationship between the rate of a chemical reaction and the activation energy is described by the Arrhenius equation:

$$R_O = A_O \exp(-\frac{E_{a,o}}{\kappa_B T}) \quad (1)$$

$$R_p = A_p \exp(-\frac{E_{a,p}}{\kappa_B T}) \quad (2)$$

where A is the pre-exponent factor; E_a is the activation energy; the subscripts *o* and *p* denote the ohmic and polarization resistance, respectively; κ_B is the Boltzmann constant and T is the absolute temperature in Kelvin.

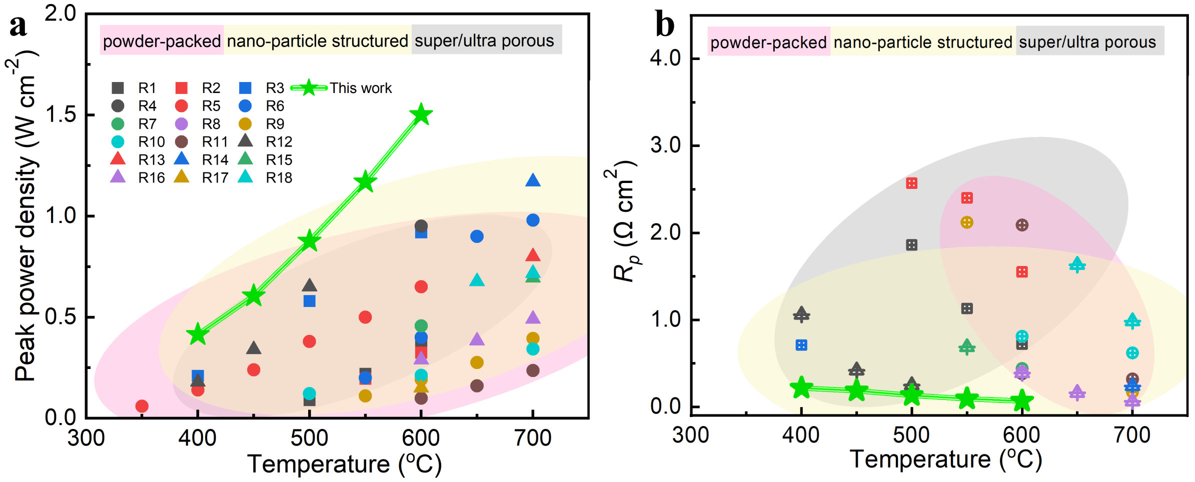

Fig. S1. Highly enhanced electrochemical performance of PCEC with nano-architecture ultra-porous (NAUP) PNC73 electrode. a Comparison on peak power density of NAUP-PNC73 (this work) in fuel cell mode with the literature results. The NAUP-PNC73 full cells are readily fabricated in this work using self-structured mesh to achieve ultra-porous configuration. Thus, another similar fabrication methods: powder-packed electrode; nano-particle electrode and super/ultra porous electrode, are used to provide a more distinct contrast. The temperature range of all conducted tests was 350-700 °C[1–18]. **b** Polarization resistance (R_p) of NAUP-PNC73 under OCV conditions in comparison with the representative results.

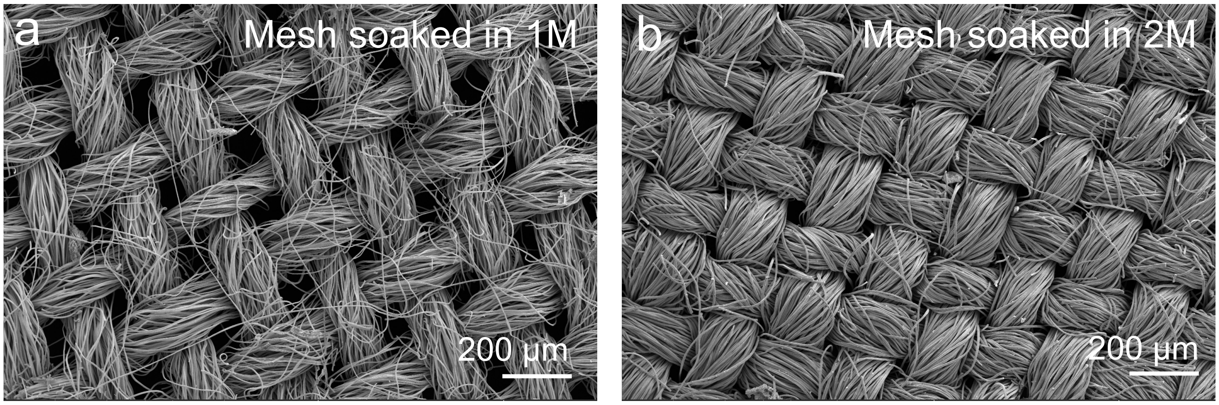

Fig. S2. SEM images of NAUP-PNC mesh-structured electrode. a Mesh soaked in 1M PNC73 solution. **b** Mesh soaked in 2M PNC73 solution.

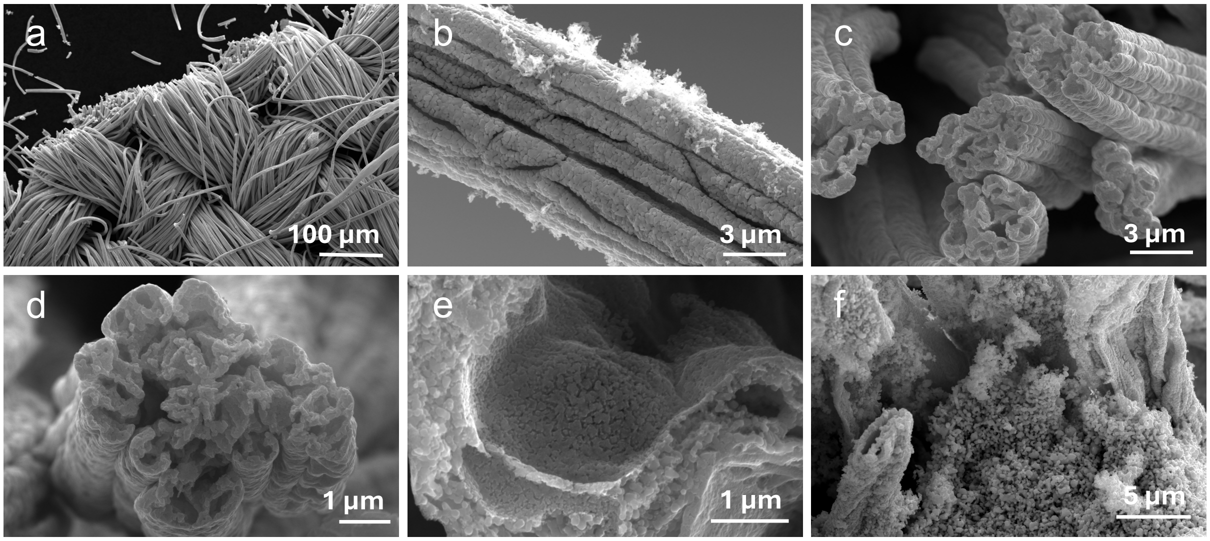

Fig. S3. SEM images of **a** NAUP-PNC mesh-structured electrode edges. **b** one NAUP-PNC mesh-structured electrode fiber. **c** cross sections of several NAUP-PNC mesh-structured electrode fibers. **d** the cross section of one NAUP-PNC mesh-structured electrode fiber. **e** the inner wall of one NAUP-PNC mesh-structured electrode fiber. **f** bonding relationship between NAUP-PNC mesh-structured electrode fibers and PNC73 ink.

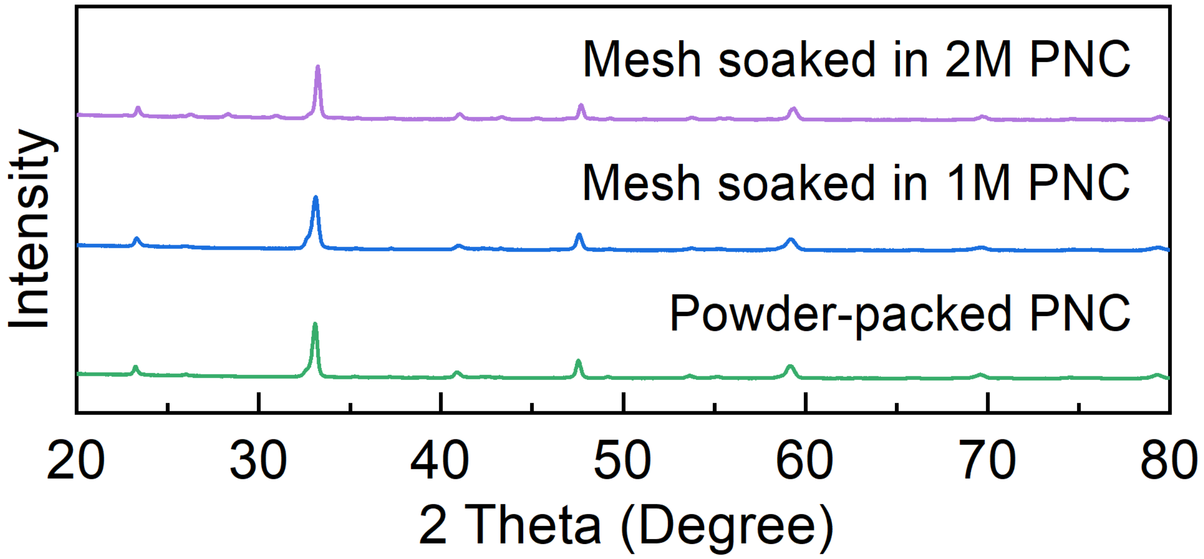

Fig. S4. XRD results of powder-packed PNC electrode. NAUP-PNC mesh-structured electrode soaked in 1M PNC73 solution and soaked in 2M PNC73 solution, respectively. All showed the pure phase.

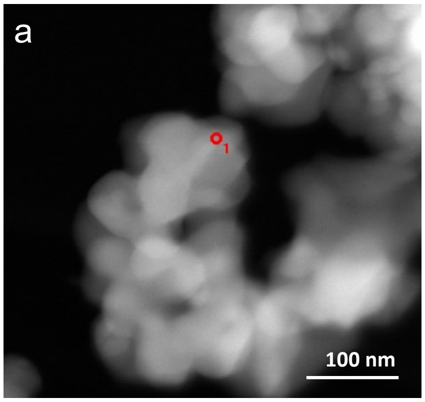

b

Element	Weight %	Atomic %	Uncert. %	Correction	k-Factor
O(K)	4.72	23.65	0.26	0.51	1.921
Co(K)	16.55	22.48	0.39	0.99	1.441
Ni(K)	11.50	15.68	0.33	0.99	1.453
Pr(L)	67.20	38.17	0.88	0.99	3.113

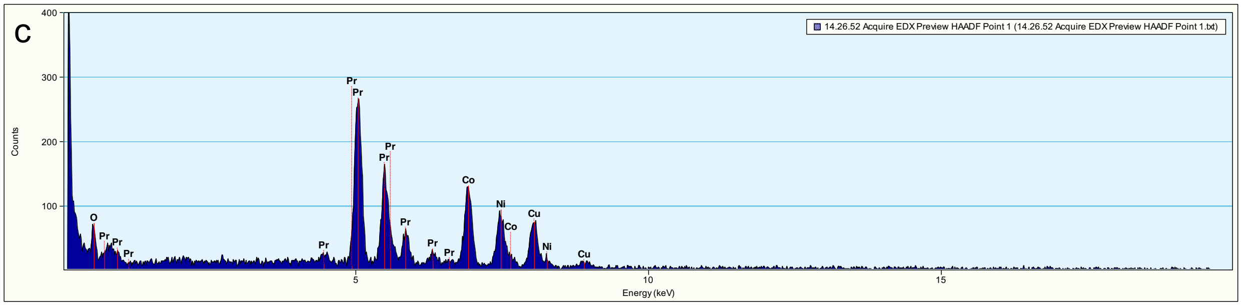

Fig. S5: TEM characterization of NAUP-PNC oxygen electrode. a HAADF image of NAUP-PNC nanoparticles. **b** Table of elemental amounts in percentages on site 1. **c** EDS spectra of HAADF site 1.

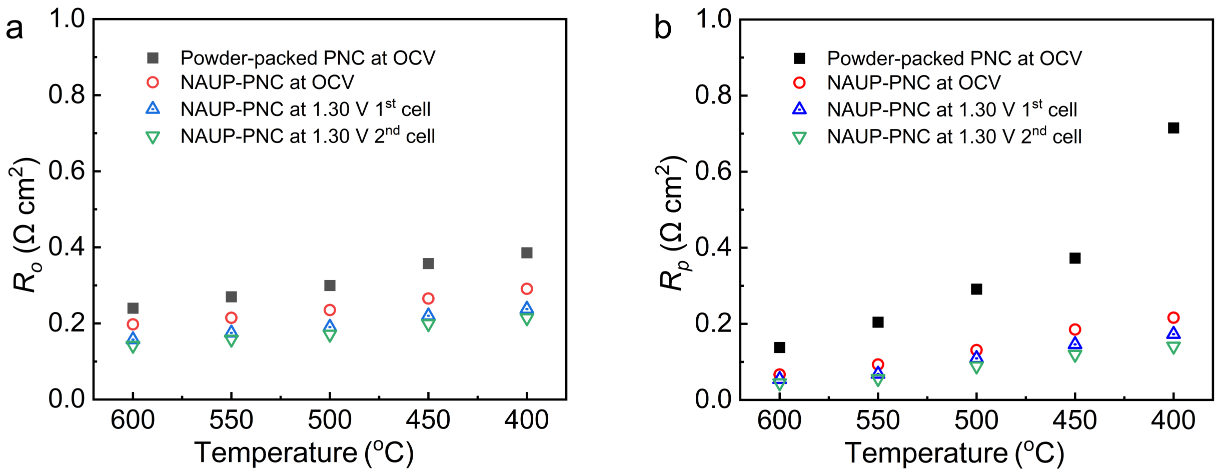

c

Powder-packed PNC at OCV	R_o (Ω cm^2)	R_p (Ω cm^2)	Standard deviation for R_p
600 ℃	0.2012	0.1767	0.00174
550 ℃	0.2641	0.3277	0.00146
500 ℃	0.3515	0.7146	0.00132
450 ℃	0.4713	1.6939	0.00142
400 ℃	0.4952	1.9105	0.00159

NAUP-PNC at OCV	R_o (Ω cm^2)	R_p (Ω cm^2)	Standard deviation for R_p
600 ℃	0.1976	0.0668	0.00155
550 ℃	0.2241	0.0840	0.00143
500 ℃	0.2373	0.1278	0.00162
450 ℃	0.2508	0.1997	0.00147
400 ℃	0.2682	0.2387	0.00167

NAUP-PNC at 1.30 V	R_o (Ω cm^2)	R_p (Ω cm^2)	Standard deviation for R_p
600 ℃	0.1577	0.0530	0.00157
550 ℃	0.1752	0.0682	0.00134
500 ℃	0.1904	0.1091	0.00144
450 ℃	0.2202	0.1460	0.00124
400 ℃	0.2376	0.1729	0.00133

Fig. S6. Comparison of R_o (a) and R_p (b) between NAUP-PNC cells and powder-packed PNC cells under both SOFC and SOEC modes at 400-600 °C. **c** Resistance value with corresponding standard deviations.

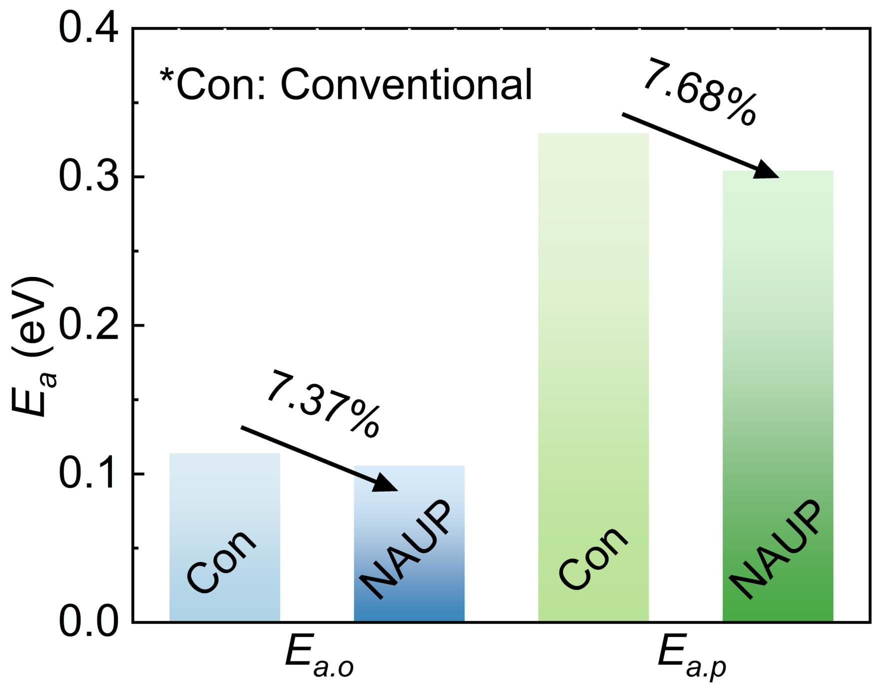

Fig. S7: Comparison between E_a from ohmic resistance and E_a from polarization resistance.

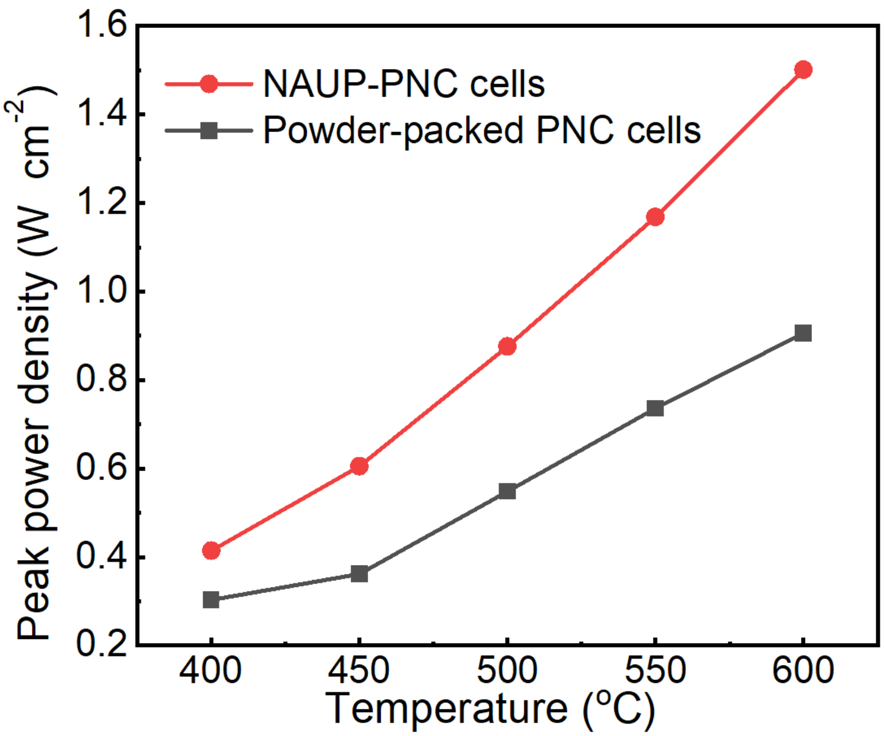

Fig. S8. Comparison of peak power density between NAUP-PNC cells and powder-packed PNC cells under SOFC mode at 400-600 ºC.

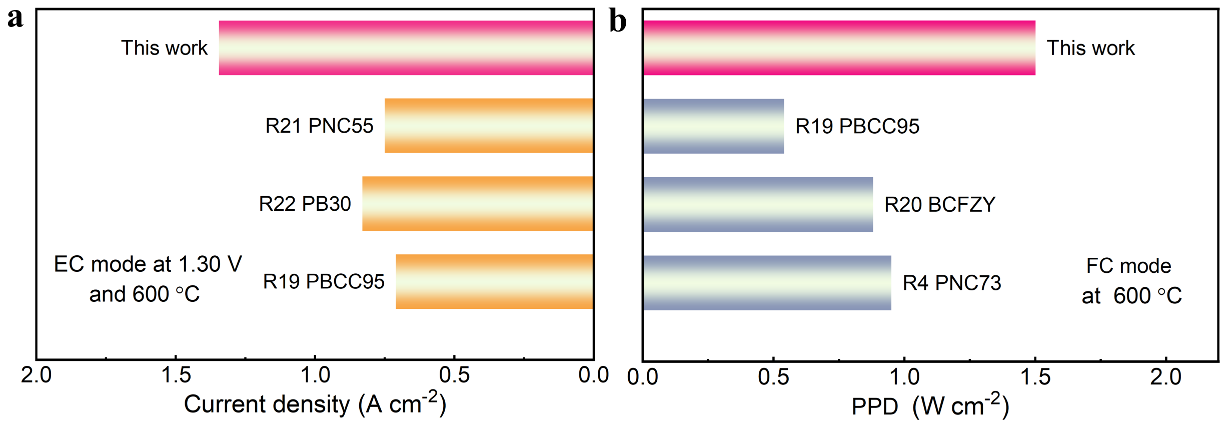

Fig. S9. Comparison of NAUP-PNC73 full cell performances in both FC and EC modes with some recent literatures[4,19–22]. BCFZY, $BaCo_{0.4}Fe_{0.4}Zr_{0.1}Y_{0.1}O_{3-\delta}$; PNC73, $PrNi_{0.7}Co_{0.3}O_{3-\delta}$; PNC55, $PrNi_{0.5}Co_{0.5}O_{3-\delta}$; PB30, $Pr_{1.7}Ba_{0.3}NiO_{4+\delta}$; PBCC95, $(PrBa_{0.8}Ca_{0.2})_{0.95}Co_2O_{6-\delta}$.

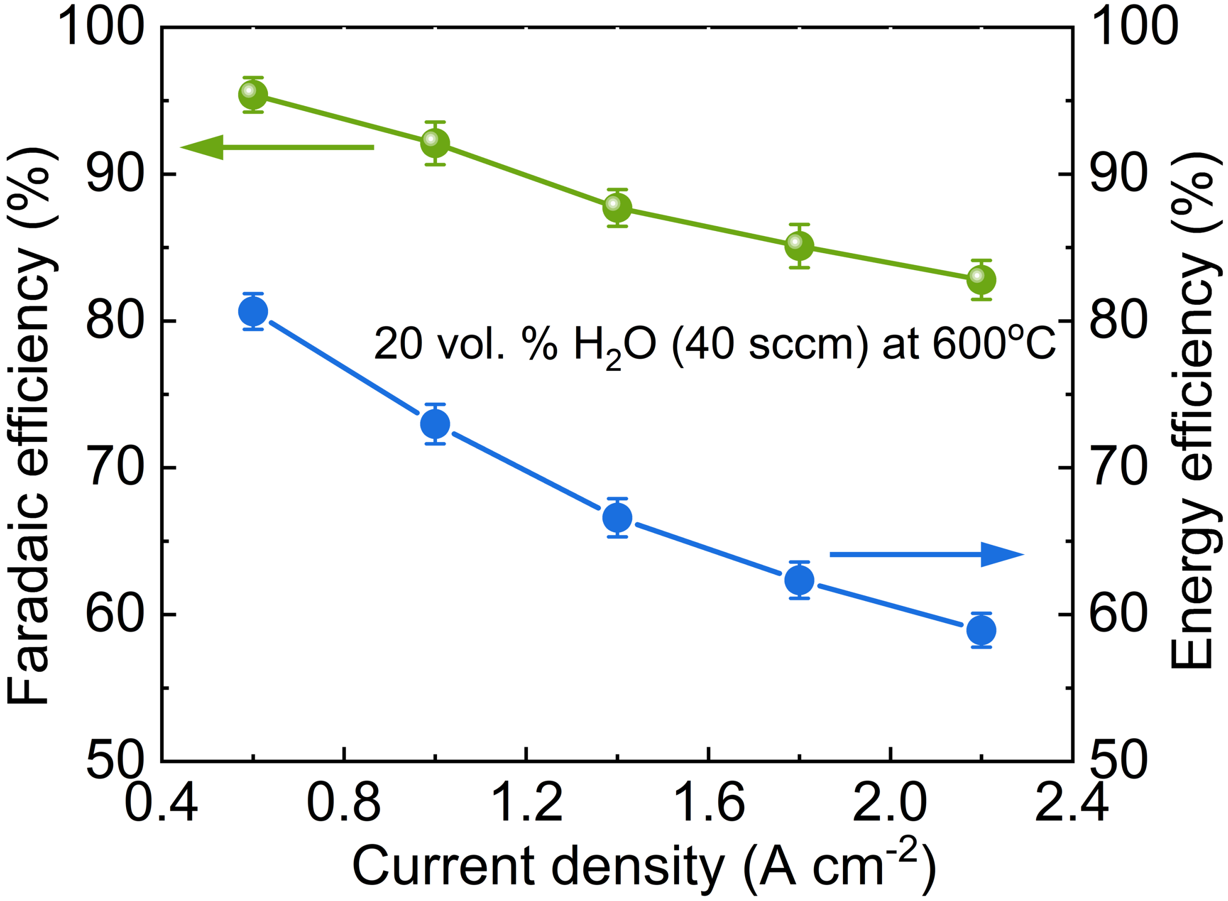

Current density (A cm^{-2})	FE (%)	Standard deviation of FE	EE (%)	Standard deviation of EE
0.60	95.4	±1.17	80.6	±1.22
1.00	92.1	±1.45	72.9	±1.34
1.40	87.7	±1.25	66.6	±1.30
1.80	85.1	±1.47	62.3	±1.24
2.20	82.8	±1.33	58.9	±1.15

Fig. S10. Faradic efficiency (FE, %) and Energy efficiency (EE, %) of NAUP-PNC full cells in electrolysis mode at 600°C (top). Table of statistical standard deviations for FE and EE (bottom). Each test was conducted for six times to get the statistical standard deviations, presented as error bars.

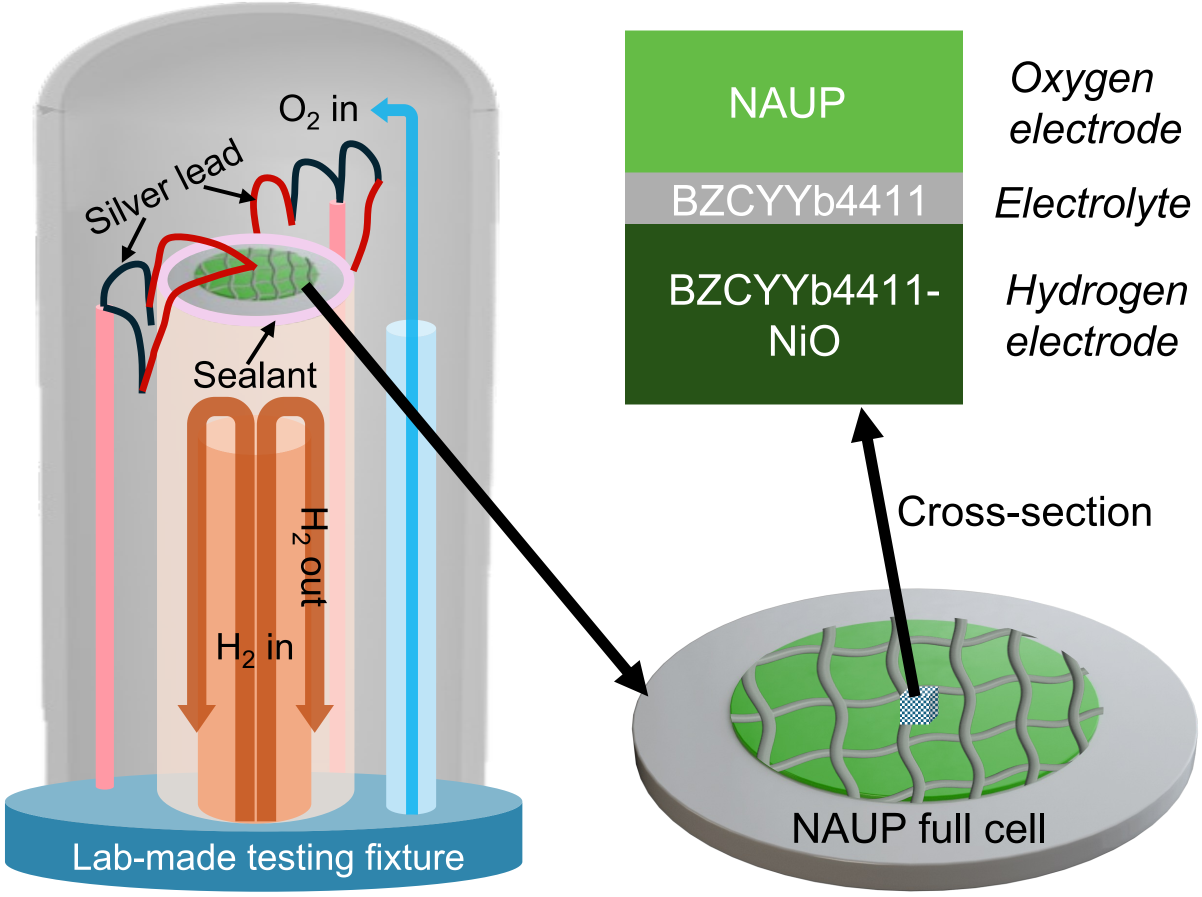

Fig. S11. Lab-made testing fixture used in this work.

PPD (W cm^{-2})	Temperature (°C)								*Experimental condition*	Fabrication
	700	650	600	550	500	450	400	350		
This work	-	-	1.50	1.17	0.88	0.61	0.41	-	*Under H_2/O_2*	NAUP
Ref. 1	-	-	0.36	0.22	0.09	-	-	-	*A steam partial pressure of 12%/Pure H_2/FC at 0.7 V*	3D self-architectured steam electrode
Ref. 2	-	-	0.33	0.20	-	-	-	-	*Cathode: 75% CO_2+25%O_2/Anode : Ar*	3D architectured electrode
Ref. 3	-	-	0.92	-	0.58	-	0.21	-	*Cathode: pure O_2/Anode: pure H_2*	3D engineering electrode
Ref. 4	-	-	0.95	-	-	-	-	-	*Wet H_2 (3% steam) on the fuel side and pure oxygen on the oxygen side*	Powder-packed electrode
Ref. 5	-	-	0.65	0.50	0.38	0.24	0.14	0.06	*Under H_2/air*	Powder-packed electrode
Ref. 6	1.30	0.90	0.40	0.20	-	-	-	-	*Under H_2/O_2*	Powder-packed electrode
Ref. 7	-	-	0.46	-	-	-	-	-	*Under H_2/O_2*	Powder-packed electrode
Ref. 8	-	-	-	-	-	-	-	-	-	Powder-packed electrode
Ref. 9	0.40	0.28	0.19	0.11	-	-	-	-	*Under H_2/air*	Powder-packed electrode
Ref. 10	0.34	-	0.21	-	0.12	-	-	-	*Humidified H_2/O_2*	Powder-packed electrode
Ref. 11	0.24	0.16	0.10	-	-	-	-	-	*Humidified hydrogen (~3% H_2O) /O_2*	Powder-packed electrode
Ref. 12	-	-	-	-	0.65	0.34	0.18	-	*Humidified hydrogen (~3% H_2O) /air*	Nano-network electrode
Ref. 13	0.80	-	-	-	-	-	-	-	*Under H_2/O_2*	Nano-tailoring electrode
Ref. 14	1.37	-	-	-	-	-	-	-	*Humidified hydrogen (~6% H_2O) /air*	Nanocatalyst electrode
Ref. 15	0.70	-	-	-	-	-	-	-	*Humidified hydrogen (~3% H_2O) /air*	Infiltrated nano-electrode
Ref. 16	0.49	0.38	0.29	-	-	-	-	-	*Humidified hydrogen (~3% H_2O) /air*	Infiltrated multiscale electrode
Ref. 17	-	-	0.15	-	-	-	-	-	*Humidified hydrogen (~3% H_2O) /air*	Nanostructured electrode
Ref. 18	0.72	0.68	-	-	-	-	-	-	*Humidified hydrogen (~3% H_2O) /air*	Nanostructured electrode

Table. S1. Comparison of peak power density between this work and other literature results.

R_p (Ω cm^2)	Temperature (^oC)								***Experimental condition***	Fabrication
	700	650	600	550	500	450	400	350		
This work	-	-	0.07	0.09	0.13	0.19	0.22	-	*Under H_2/O_2 /at OCV*	NAUP
Ref. 1	-	-	0.72	1.13	1.86	-	-	-	*A steam partial pressure of 12%/Pure H_2/FC at OCV*	3D self-architectured steam electrode
Ref. 2	-	-	1.55	2.10	2.57	-	-	-	*Cathode: 75% CO_2+25%O_2/Anode : Ar/at OCV*	3D architectured electrode
Ref. 3	-	-	-	-	-	-	0.71	-	*Cathode: pure O_2/Anode: pure H_2 /at OCV*	3D engineering electrode
Ref. 4	-	-	0.38	-	-	-	-	-	*Wet O_2 (3% steam) at OCV*	Powder-packed electrode
Ref. 5	-	-	-	-	-	-	-	-	-	Powder-packed electrode
Ref. 6	-	-	-	-	-	-	-	-	-	Powder-packed electrode
Ref. 7	-	-	0.44	-	-	-	-	-	*Under H_2/O_2 /at OCV*	Powder-packed electrode
Ref. 8	0.31	-	0.40	-	-	-	-	-	*Steam in anode: 0.4 atm*	Powder-packed electrode
Ref. 9	0.17	-	-	2.12	-	-	-	-	*Under H_2/air /at OCV*	Powder-packed electrode
Ref. 10	0.62	-	0.81	-	-	-	-	-	*Humidified H_2/O_2 /at OCV*	Powder-packed electrode
Ref. 11	0.32	-	2.09	-	-	-	-	-	*Humidified hydrogen (~3% H_2O) /O_2/at OCV*	Powder-packed electrode
Ref. 12	-	-	-	-	0.25	0.42	1.06	-	*Humidified hydrogen (~3% H_2O) /air /at OCV*	Nano-network electrode
Ref. 13	-	-	-	-	-	-	-	-	-	Nano-tailoring electrode
Ref. 14	0.24	-	-	-	-	-	-	-	*Humidified hydrogen (~6% H_2O) /air /at OCV*	Nanocatalyst electrode
Ref. 15	-	-	-	0.67	-	-	-	-	*Humidified hydrogen (~3% H_2O) /air /at OCV*	Infiltrated nano-electrode
Ref. 16	0.06	0.16	0.39	-	-	-	-	-	*Humidified hydrogen (~3% H_2O) /air /at OCV*	Infiltrated multiscale electrode
Ref. 17	-	-	-	-	-	-	-	-	-	Nanostructured electrode
Ref. 18	0.99	1.63	-	-	-	-	-	-	*Humidified hydrogen (~3% H_2O) /air /at OCV*	Nanostructured electrode

Table. S2. Comparison of R_p between this work and other literature results.

R_o	E_a (eV)	Standard deviation
Powder-packed PNC at OCV	0.1139	±0.01399
NAUP-PNC at OCV	0.1055	±0.01197
NAUP-PNC at 1.30 V 1st cell	0.1059	±0.01323
NAUP-PNC at 1.30 V 2nd cell	0.1064	±0.01378

Table. S3. Calculated $E_{a,o}$ with standard errors of NAUP-PNC cells and powder-packed PNC cells under both SOFC and SOEC modes.

R_p	E_a (eV)	Standard deviation
Powder-packed PNC at OCV	0.32935	±0.01807
NAUP-PNC at OCV	0.30406	±0.01371
NAUP-PNC at 1.30 V 1st cell	0.30768	±0.01742
NAUP-PNC at 1.30 V 2nd cell	0.30817	±0.01728

Table. S4. Calculated $E_{a,p}$ with standard errors of NAUP-PNC cells and powder-packed PNC cells under both SOFC and SOEC modes.

NAUP-PNC cells					
Temperature (°C)	400	450	500	550	600
Peak power density (W cm^{-2})	0.4140	0.6055	0.8762	1.1684	1.5012

Powder-packed PNC cells					
Temperature (°C)	400	450	500	550	600
Peak power density (W cm^{-2})	0.3031	0.3619	0.5481	0.7364	0.9056

Table. S5. Comparison of peak power density between NAUP-PNC cells (top) and powder-packed PNC cells (bottom) under SOFC mode at 400-600 °C.

NAUP-PNC ells					
Temperature (°C)	400	450	500	550	600
Current density (A cm^{-2})	-0.6512	-1.5245	-2.4985	-3.8627	-5.0423

Powder-packed PNC cells					
Temperature (°C)	400	450	500	550	600
Current density (A cm^{-2})	-0.4932	-1.1012	-1.5321	-2.6021	-3.3605

Table. S6. Comparison of current density of NAUP-PNC cells (top) and powder-packed PNC cells (bottom) under SOEC mode at 400-600 °C.

Relative humidity (vol.% H_2O)	3	10	20	30
Current density (A cm^{-2})	-4.0265	-4.5124	-5.0397	-4.7397

Gas flow rate (sccm)	10	20	40	60
Current density (A cm^{-2})	-4.2151	-4.6001	-5.0258	-4.8225

Table. S7. Comparison of current density of NAUP-PNC cells in terms of relative humidity (top) and gas flow rate (bottom) under SOEC mode at 600 ºC.

PPD (W cm^{-2})				
Ref.	R4<br>PNC73	R20<br>BCFZY	R19<br>PBCC95	This work
Conditions	H_2-Humidified O_2 (50% H_2O)	Humidified H_2 (3% H_2O) -Humidified O_2 (3% H_2O)	Humidified H_2 (3% H_2O) -O_2	H_2-Humidified O_2 (3% H_2O)
PPD (W cm^{-2})	0.95	0.88	0.54	1.50

CD at 1.30V (A cm^{-2})				
Ref.	R22<br>PB30	R19<br>PBCC95	R21<br>PNC55	This work
Conditions	H_2-Humidified O_2 (60% H_2O)	Humidified H_2 (3% H_2O) -Humidified O_2 (20% H_2O)	Humidified H_2 (3% H_2O) -Humidified O_2 (50% H_2O)	H_2-Humidified O_2 (20% H_2O)
CD at 1.30V (A cm^{-2})	0.83	0.71	0.75	1.35

Table. S8. Comparison of peak power density and current density for the reference data in Fig. S9.

Xth	R_o (Ω cm^2)	R_t (Ω cm^2)	R_p (Ω cm^2)
1st	0.1577	0.2099	0.05220
2nd	0.1579	0.2084	0.05055
3rd	0.1582	0.2098	0.05158
4th	0.1586	0.2116	0.05304
5th	0.1592	0.2095	0.05029
6th	0.1583	0.2085	0.05026
7th	0.1572	0.2093	0.05214
8th	0.1576	0.2084	0.05083
9th	0.1574	0.2081	0.05061
10th	0.1558	0.2091	0.05330
11th	0.1554	0.2099	0.05450
12th	0.1571	0.2095	0.05235
13th	0.1563	0.2100	0.05368
14th	0.1535	0.2095	0.05599
15th	0.1535	0.2095	0.05596
16th	0.1548	0.2095	0.05465
17th	0.1524	0.2109	0.05851
18th	0.1547	0.2079	0.05313
19th	0.1586	0.2116	0.05304
20th	0.1554	0.2087	0.05336

Table. S9. Results of R_o, R_t and R_p of NAUP-PNC cells at 1.30 V and 600 ºC for 20 cycles.

Time (h)	Current density (A cm^{-2})			Mean %
	-Value	Difference	% of degradation	
0	1.2889			
10	1.2763	0.0126	0.98	
20	1.2632	0.0131	1.03	
30	1.2499	0.0133	1.05	1.03
40	1.2369	0.0130	1.04	
50	1.2239	0.0130	1.05	
Altering applied voltage from 1.30 V to 1.40 V				
50	1.9958			
60	1.9729	0.0230	1.15	
70	1.9482	0.0247	1.25	
80	1.9242	0.0240	1.23	1.24
90	1.9002	0.0241	1.25	
100	1.8753	0.0249	1.31	

Table. S10. % of degradation calculation on current density of NAUP-PNC cells at 1.30 V/1.40 V and 600 ºC for 100 hours.

Time (h)	Current density ($A\ cm^{-2}$)			Mean %
	Mode	Standard deviation	% of degradation	
0-10		0.02036	1.69	
20-30		0.02168	1.83	
40-50	SOEC	0.02244	1.93	1.87
60-70		0.02144	1.88	
80-90		0.02204	1.97	
10-20		0.02823	1.48	
30-40		0.02969	1.58	
50-60	SOFC	0.02977	1.61	1.55
70-80		0.02893	1.59	
90-100		0.02668	1.49	

Table. S11. % of degradation calculation on current density of NAUP-PNC cells for reversible tests at 600 ºC for 100 hours.

*X*th	Current density (A cm^{-2})			Mean %
	Voltage (V)	Standard deviation	% of degradation	
1		0.05634	1.85	
2		0.05410	1.81	
3		0.05605	1.91	
4		0.05239	1.82	
5		0.05257	1.86	
6		0.05104	1.84	
7	1.50	0.05935	2.18	1.94
8		0.05620	2.11	
9		0.05423	2.08	
10		0.04902	1.92	
11		0.04732	1.89	
12		0.04889	1.99	
13		0.04623	1.92	

Table. S12. % of degradation calculation on current density of NAUP-PNC cells for transient tests (step-voltage based) at 1.50 V and 600 ºC.

*X*th	Current density (A cm^{-2})			Mean %
	Voltage (V)	Standard deviation	% of degradation	
1&2		0.02270	1.35	
3&4		0.02273	1.37	
5&6		0.02176	1.33	
7&8		0.02164	1.34	
9&10		0.02023	1.27	
11&12		0.01903	1.21	
13&14	1.35	0.01989	1.28	1.28
15&16		0.01856	1.21	
17&19		0.01955	1.29	
19&20		0.01959	1.31	
21&22		0.01757	1.19	
23&24		0.01823	1.25	

Table. S13. % of degradation calculation on current density of NAUP-PNC cells for transient tests (step-voltage based) at 1.35 V and 600 ºC.

*X*th	Current density (A cm^{-2})			Mean %
	Voltage (V)	Standard deviation	% of degradation	
1	1.20	0.00327	0.45	0.45
2		0.00304	0.42	
3		0.00303	0.42	
4		0.00352	0.49	
5		0.00343	0.48	
6		0.00313	0.44	
7		0.00318	0.45	
8		0.00303	0.43	
9		0.00358	0.51	
10		0.00272	0.39	
11		0.00306	0.44	
12		0.00311	0.45	

Table. S14. % of degradation calculation on current density of NAUP-PNC cells for transient tests (step-voltage based) at 1.20 V and 600 ºC.

*X*th	Current density (A cm^{-2})			Mean %
	Voltage (V)	Standard deviation	% of degradation	
1		0.00722	0.24	
2		0.04665	1.56	
3		0.00393	0.13	
4		0.03728	1.26	
5		0.03791	1.30	
6		0.01210	0.41	
7		0.05000	1.69	
8	1.50	0.03888	1.33	1.07
9		0.03888	1.32	
10		0.07500	2.57	
11		0.04155	1.39	
12		0.03321	1.13	
13		0.00118	0.04	
14		0.01784	0.60	
15		0.03333	1.12	

Table. S15. % of degradation calculation on current density of NAUP-PNC cells for transient tests (time-interval based) at 1.50 V and 600 ºC.

*X*th	Current density (A cm^{-2})			Mean %
	Voltage (V)	Standard deviation	% of degradation	
1	1.20	0.00747	0.75	4.11
2		0.00333	0.33	
3		0.02334	2.34	
4		0.00666	0.68	
5		0.06167	6.38	
6		0.08500	9.39	
7		0.00500	0.51	
8		0.06833	6.87	
9		0.05000	5.40	
10		0.01333	1.36	
11		0.08500	8.59	
12		0.05333	5.89	
13		0.04000	4.17	
14		0.08666	8.68	
15		0.04000	4.39	
16		0.02916	3.06	

Table. S16. % of degradation calculation on current density of NAUP-PNC cells for transient tests (time-interval based) at 1.20 V and 600 ºC.

Voltage (V)	% of degradation	Mean %
1.50 V	1.07	2.59
1.20 V	4.11	

Table. S17. Mean % of degradation calculation on current density of NAUP-PNC cells for transient tests (time-interval based) at 600 ºC.

*X*th	CD at SOFC (A cm^2)	CD at SOEC (A cm^2)	Temperature (°C)
1st	4.68322	-4.87872	600
2nd	3.34564	-2.94545	550
3rd	3.09026	-2.40625	500
4th	3.28819	-2.89412	550
5th	4.58335	-4.78633	600
6th	3.21531	-2.76239	550
7th	2.92903	-1.90668	500
8th	2.95254	-2.49223	550
9th	4.4722	-4.78633	600
10th	2.78259	-2.32329	550
11th	2.76781	-1.7373	500
12th	2.66128	-2.20302	550
13th	4.47308	-4.67502	600
14th	2.52947	-2.06924	550
15th	2.40252	-1.1122	500

Table. S18. Results of maximum current density of NAUP-PNC cells for thermal cycling tests.

Xth	R_o (Ω cm^2)	R_t (Ω cm^2)	R_p (Ω cm^2)	Standard deviation for R_p	Temperature (°C)
1st	0.16555	0.22275	0.05720	0.00134	600
2nd	0.18043	0.25075	0.07032	0.00115	550
3rd	0.19417	0.30703	0.11286	0.00107	500
4th	0.18049	0.25687	0.07638	0.00129	550
5th	0.16531	0.22604	0.06073	0.00135	600
6th	0.1806	0.2615	0.08090	0.00147	550
7th	0.19399	0.31509	0.12110	0.00151	500
8th	0.18176	0.26166	0.07990	0.00147	550
9th	0.16625	0.22853	0.06228	0.00132	600
10th	0.18162	0.2627	0.08108	0.00132	550
11th	0.19703	0.32481	0.12778	0.00128	500
12th	0.18127	0.26595	0.08468	0.00121	550
13th	0.16689	0.23154	0.06465	0.00145	600
14th	0.18162	0.26827	0.08665	0.00133	550
15th	0.19725	0.33375	0.13650	0.00129	500

Table. S19. Results of R_o, R_t and R_p of NAUP-PNC cells at 1.30 V for thermal cycling tests.

References

1. Wu, W. *et al.* 3D Self-Architectured Steam Electrode Enabled Efficient and Durable Hydrogen Production in a Proton-Conducting Solid Oxide Electrolysis Cell at Temperatures Lower Than 600 °C. *Advanced Science* **5**, 1800360 (2018).
2. Wu, W., Zhang, Y., Ding, D. & He, T. A High-Performing Direct Carbon Fuel Cell with a 3D Architectured Anode Operated Below 600 °C. *Advanced Materials* **30**, 1704745 (2018).
3. Bian, W. *et al.* Regulation of Cathode Mass and Charge Transfer by Structural 3D Engineering for Protonic Ceramic Fuel Cell at 400 °C. *Adv Funct Materials* **31**, 2102907 (2021).
4. Tang, W. *et al.* An Unbalanced Battle in Excellence: Revealing Effect of Ni/Co Occupancy on Water Splitting and Oxygen Reduction Reactions in Triple-Conducting Oxides for Protonic Ceramic Electrochemical Cells. *Small* **18**, 2201953 (2022).
5. Duan, C. *et al.* Readily processed protonic ceramic fuel cells with high performance at low temperatures. *Science* **349**, 1321–1326 (2015).
6. Fang, L. *et al.* Boosting the performance of reversible solid oxide electrochemical cells with a novel hybrid oxygen electrode, $Pr_{1.39}Ba_{0.14}Sr_{0.53}Co_{1.48}Fe_{0.76}O_{6-\delta}$-$Ba_{0.66}Sr_{0.34}CoO_{3-\delta}$. *J. Mater. Chem. A* **11**, 21251–21262 (2023).
7. Zheng, S., Bian, W. & Ding, H. A robust protonic ceramic fuel cell with a triple conducting oxygen electrode under accelerated stress tests. *Mater. Adv.* **5**, 2296–2305 (2024).
8. Li, W. *et al.* High performing triple-conductive $Pr_2NiO_{4+\delta}$ anode for proton-conducting steam solid oxide electrolysis cell. *J. Mater. Chem. A* **6**, 18057–18066 (2018).
9. Zhang, X., Zhang, H. & Liu, X. High performance La2NiO4+-infiltrated (La0.6Sr0.4)0.995Co0.2Fe0.8O3− cathode for solid oxide fuel cells. *Journal of Power Sources* **269**, 412–417 (2014).

10. Bi, L. *et al.* Indium as an ideal functional dopant for a proton-conducting solid oxide fuel cell. *International Journal of Hydrogen Energy* **34**, 2421–2425 (2009).

11. Tao, Z., Zhu, Z., Wang, H. & Liu, W. A stable BaCeO3-based proton conductor for intermediate-temperature solid oxide fuel cells. *Journal of Power Sources* **195**, 3481–3484 (2010).

12. Chen, Y. *et al.* Low temperature solid oxide fuel cells with hierarchically porous cathode nano-network. *Nano Energy* **8**, 25–33 (2014).

13. Ishfaq, H. A. *et al.* Boosting performance of the solid oxide fuel cell by facile nano-tailoring of La0.6Sr0.4CoO3-δ cathode. *International Journal of Hydrogen Energy* **47**, 37587–37598 (2022).

14. Namgung, Y., Hong, J., Kumar, A., Lim, D.-K. & Song, S.-J. One step infiltration induced multi-cation oxide nanocatalyst for load proof SOFC application. *Applied Catalysis B: Environmental* **267**, 118374 (2020).

15. Lou, X., Wang, S., Liu, Z., Yang, L. & Liu, M. Improving La0.6Sr0.4Co0.2Fe0.8O3−δ cathode performance by infiltration of a Sm0.5Sr0.5CoO3−δ coating. *Solid State Ionics* **180**, 1285–1289 (2009).

16. Zhao, F., Liu, Q., Wang, S. & Chen, F. Infiltrated multiscale porous cathode for proton-conducting solid oxide fuel cells. *Journal of Power Sources* **196**, 8544–8548 (2011).

17. Lu, C., Sholklapper, T. Z., Jacobson, C. P., Visco, S. J. & De Jonghe, L. C. LSM-YSZ Cathodes with Reaction-Infiltrated Nanoparticles. *J. Electrochem. Soc.* **153**, A1115 (2006).

18. Rehman, S. U. *et al.* Nano-fabrication of a high-performance LaNiO3 cathode for solid oxide fuel cells using an electrochemical route. *Journal of Power Sources* **429**, 97–104 (2019).

19. Tang, W. *et al.* Understanding of A-site deficiency in layered perovskites: promotion of dual reaction kinetics for water oxidation and oxygen reduction in protonic ceramic electrochemical cells. *J. Mater. Chem. A* **8**, 14600–14608 (2020).

20. Kim, D. *et al.* High-Performance Protonic Ceramic Electrochemical Cells. *ACS Energy Lett.* **7**, 2393–2400 (2022).

21. Rajendran, S. *et al.* Tri-Doped $BaCeO_3$ –$BaZrO_3$ as a Chemically Stable Electrolyte with High Proton-Conductivity for Intermediate Temperature Solid Oxide Electrolysis Cells (SOECs). *ACS Appl. Mater. Interfaces* **12**, 38275–38284 (2020).

22. Li, W. *et al.* Layer-structured triple-conducting electrocatalyst for water-splitting in protonic ceramic electrolysis cells: Conductivities vs. activity. *Journal of Power Sources* **495**, 229764 (2021).
